# Progress of Transposon Vector System for Production of Recombinant Therapeutic Proteins in Mammalian Cells

**DOI:** 10.3389/fbioe.2022.879222

**Published:** 2022-05-04

**Authors:** Mian Wei, Chun-Liu Mi, Chang-Qin Jing, Tian-Yun Wang

**Affiliations:** ^1^ School of Life Science and Technology, Xinxiang Medical University, Xinxiang, China; ^2^ International Joint Research Laboratory for Recombinant Pharmaceutical Protein Expression System of Henan, Xinxiang, China

**Keywords:** recombinant therapeutic protein, mammalian cells, transposon vector, transposase, PiggyBac

## Abstract

In recent years, mammalian cells have become the primary host cells for the production of recombinant therapeutic proteins (RTPs). Despite that the expression of RTPs in mammalian cells can be improved by directly optimizing or engineering the expression vectors, it is still influenced by the low stability and efficiency of gene integration. Transposons are mobile genetic elements that can be inserted and cleaved within the genome and can change their inserting position. The transposon vector system can be applied to establish a stable pool of cells with high efficiency in RTPs production through facilitating the integration of gene of interest into transcriptionally active sites under screening pressure. Here, the structure and optimization of transposon vector system and its application in expressing RTPs at high level in mammalian cells are reviewed.

## 1 Introduction

Seventy-one new biological drugs have been approved and launched into the market from 2014 to 2018, of which 62 are recombinant therapeutic proteins (RTPs) (Walsh 2018). Recombinant proteins are produced in heterologous cells using genetic engineering techniques by obtaining the gene of interest (GOI), constructing the expression vector, and expressing the protein of interest in the host cell. The trend of using mammalian cell lines in RTPs production has accelerated dramatically in recent years, 84% of approved RTPs were produced using mammalian cells in 2018 (Walsh 2018; Tschorn et al., 2020). The protein produced from Chinese hamster ovary (CHO) cells have similar post-translational modification (PTM) system to those of mammalian cell, therefore approximately 70% of the approved recombinant therapeutic protein (antibody) are produced in CHO cells. Nowadays, CHO cells have become the most commonly used mammalian cell expression system (Rajendra et al., 2017; Shin et al., 2021).

To meet the increasing demand in biopharmaceutical market, it is necessary that innovating the production process with a higher production capacity, high quality product, and reducing production costs. Vector frequently used for gene delivery are largely divided into viral and non-viral vectors. Viral vectors can infect a target cell naturally and effectively, with no additional reagent or equipment required, but it may elicit unwanted cellular consequences and the maximum vector size of viral vectors is restricted, hampering gene delivery of larger genes. In contrast, nonviral DNA plasmid-based vectors are largely nonimmunogenic and can carry large amounts of DNA, but the low transfection and poor integration rates are their major problems (Hackett et al., 2010; Chen et al., 2018). Although optimization of expression vector and cell line are the effective methods to improve transgene expression in CHO cells (Jazayeri et al., 2018), most of these methods mostly aim to promote transcription of the GOI and cannot address the problem of inefficient stable integration, with only about 1–5 copies of GOI randomly integrated into the host cell genome (Chusainow et al., 2009; Kolacsek et al., 2014). The construction of cell lines for expressing recombinant proteins by relying on random integration of the GOI might result in unpredictable and highly variable expression levels of the GOI (Matasci et al., 2011a). The transposon vector system could integrate GOI into transcription active sites more efficiently and promote the expression of recombinant proteins in stable cell pools and monoclonal cell lines (Balasubramanian et al., 2016), and has been applied in the construction of mammalian cell lines for large-scale production of recombinant proteins (Matasci et al., 2011b). Mammalian cells and expression vectors.

## 2 Mammalian Cells and Expression Vectors

### 2.1 Mammalian Cells

The main recombinant protein expression systems contain prokaryotic, yeast, insect, and mammalian cell expression systems. For the expression of the complex recombinant proteins with high molecule weight, proper folding and post-translational modifications are required to display their biological activities due to their complex structure. Therefore, mammalian cells mainly including CHO cells and human embryonic kidney (HEK293) cells, have become the main expression hosts for RTPs production due to their PTM system.

Compared with prokaryotic, yeast and insect cells, CHO cells have the following advantages (Khan 2013): 1) CHO cells can grow under the adhere and suspension state with high cell density, which are suitable for large-scale industrial production (Lai et al., 2013): 2) CHO cells are less sensitive to human virus infection (O'Flaherty et al., 2020): 3) The expressed proteins have high similarity with natural proteins in terms of molecular structure, physicochemical properties and biological functions, and the glycosylation is also more similar to that of human-derived cells due to the lack of immunogenic α-galactose epitopes (Ghaderi et al., 2012): 4) CHO cells are fibroblasts with low endogenous protein secretion, which is beneficial to the isolation and purification of recombinant proteins. (Mohan et al., 2008; Li et al., 2018). In addition, by constructing DNA methyltransferase-deficient CHO cells, the stability of expression for the recombinant protein can be enhanced by inactivating DNA methylation (Jia et al., 2018; Wang et al., 2019).

Besides, the transformed cell line HEK293 derived from human embryos have unparalleled advantages in comparison to other engineered cells in recombinant protein expression, 1) high efficiency in transfection (Dalton and Barton 2014); 2) no potential risk of rodent virus infection; 3) high ability in propeptide hydrolysis and unique γ-carboxylation modifications; 4) The glycosylation and other PTMs are fully consistent with human proteins, which can make the RTPs have the same biological activity as human cells. (Dumont et al., 2016).

In addition to CHO and HEK293, other mammalian cells used to produce RTPs include human embryonic retina cells, a suspension-adapted Madin-Daby canine kidney cells, African green monkey kidney fibroblast cell, murine myeloma cell, baby hamster kidney cells and others (Butler and Meneses-Acosta 2012; Zhu 2012). Kuczewski et al. reported that the expression of monoclonal antibodies with human embryonic retina cells, cell densities approaching 1 × 10^8^ cells/mL, titers of secreted protein levels of 8 g/L in fed-batch or 25 g/L in perfusion cultures (Kuczewski et al., 2011).

### 2.2 Expression Vectors

**FIGURE 1:**
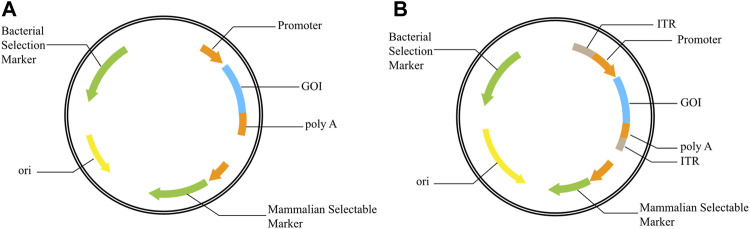
Schematic representation of vectors **(A)**: Basic structure of expression vector; **(B)**: Basic structure of transposon vector, ITR added on both sides of GOI. GOI: gene of interest; ITR: inverted terminal repeat; ori: origin of replication.

In the process of producing recombinant protein in mammalian cells, expression vector plays an important role in the expression level and stability of recombinant protein. The constituent elements of an effective mammalian cell expression vector mainly include origin of replication, promoters, screening markers, enhancers, Poly A signals, antibiotic resistance genes, expression enhancing elements, and GOI (Goetze et al., 2005) (Figure 1A). Some polycistron vectors also include internal ribosome entry sites (IRES) or Furin-2A. Efficient expression vectors depend not only on any separate elements but also on their crosstalk and interaction. An appropriate combination of expression vector elements can improve the expression of recombinant proteins, overcome gene silencing, and increase the stability of transgene expression. At present, there have been reports on matrix attachment regions (MARs) (Gao et al., 2019; Jia et al., 2019; Li et al., 2019), ubiquitous chromatin opening elements (Saunders et al., 2015), cis-acting factors (Wang et al., 2018), stabilizing Anti Repressor elements (Hoeksema et al., 2011), introns (Osabe et al., 2017), internal ribosome entry sites (IRES) (Chai et al., 2018), exons (Lu et al., 2017) and promoters (Wang et al., 2017; Yi et al., 2020), and found that these elements can improve the expression of recombinant proteins to some extent.

However, expression vectors are usually integrated into the host cell genome at random and the expression level of recombinant proteins depends on the integration site on the chromosome, but most of the genomic loci are transcriptionally repressive, resulting in some transgenic sequences cannot be expressed efficiently (Wang and Guo 2020). Thus, it is impossible to further increase the expression level even with optimized vectors. Moreover, the bacterial-associated elements, such as plasmid replication starters and antibiotic resistance genes, had been recognized as foreign sequences by the host cell and lead to methylation silencing of neighboring promoters or enhancers (Riu et al., 2007) which greatly inhibited protein production.

Studies have demonstrated that transposon vector systems could integrate GOI into transcriptionally active sites and improve positive integration efficiency (Wilson et al., 2007; Galvan et al., 2009; Grabundzija et al., 2010; Meir et al., 2011). In addition, only the promoter and GOI between the upstream and downstream inverted terminal repeat (ITR) of the vector can be transposed into the host cell genome under the action of transposase, effectively avoiding the integration of other bacterial related elements and allowing normal expression of GOI (Figure 1B).

## 3 Transposon Systems

### 3.1 Transposon Vector Structure

**FIGURE 2:**
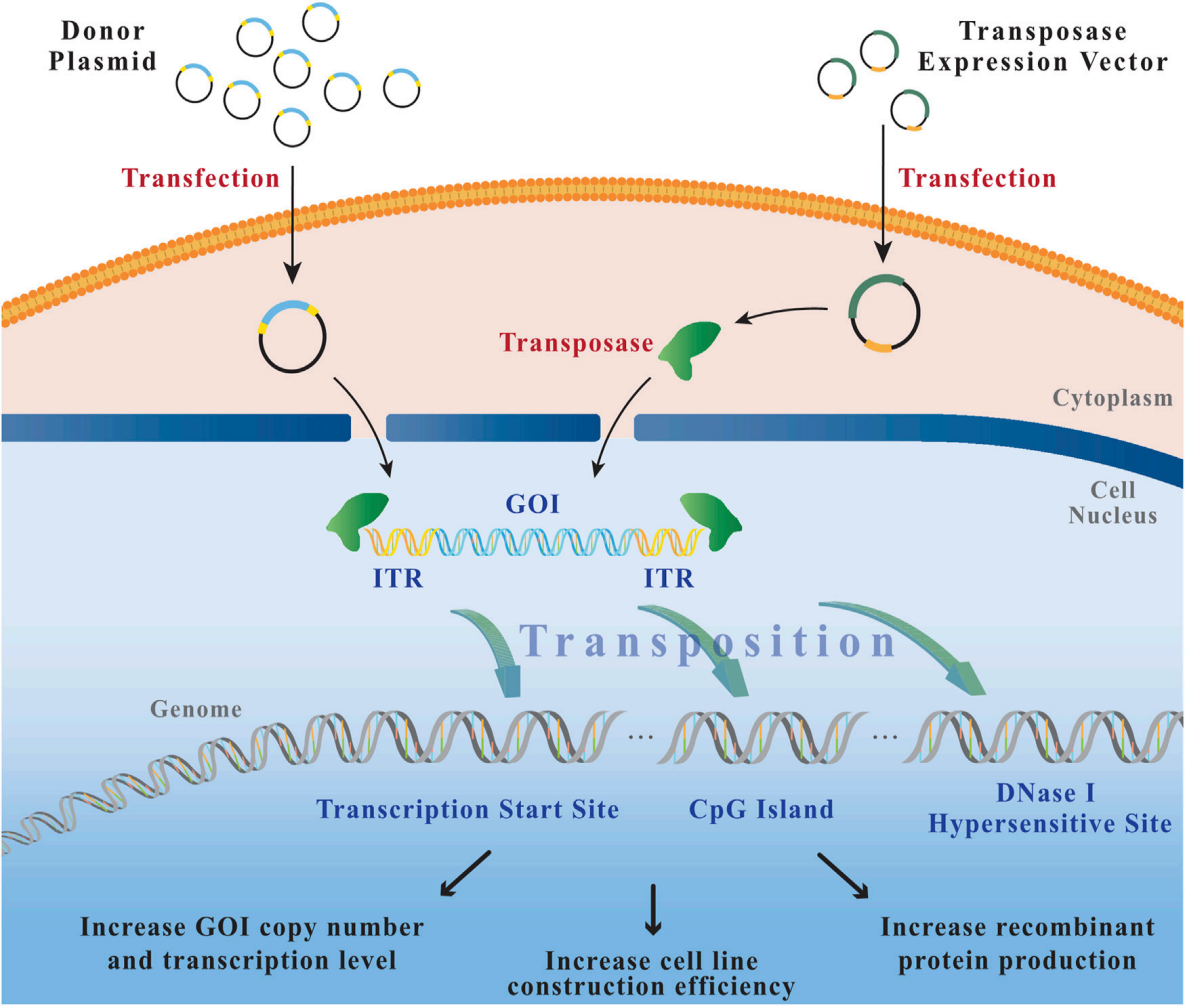
The process of transposition. After co-transfection of the donor plasmid (transposon) and the helper plasmid (transposase), the transposase binds to the ITRs of the donor plasmid, cuts the transposon vector fragment from the plasmid backbone, and integrates it into the host cell genome through a “cut-and-paste” mechanism. GOI: gene of interest; ITR: inverted terminal repeat.

The transposon is a mobile DNA element capable of transposing within genomes, can even by translocation to transpose between genomes (Sandoval-Villegas et al., 2021), which is an ideal vehicle for transporting GOI into and out of the host genome (Meir et al., 2013). Basically, all DNA transposons consist of a transposase gene and ITR sequences (Munoz-Lopez and Garcia-Perez 2010). Transposases recognize specific short target sequences located in ITRs, called directed repeat sequences (DRS). Upon binding, transposase shears transposon sequences from the genomic DNA of the host cell. Transposase cuts the genomic DNA at a new site and inserts transposon fragments. The ligation of open DNA ends is accomplished by the cell-critical factor of the non-homologous end joining pathway in the double-strand break repair system (Mátés et al., 2007). Thus, this so-called translocation occurs through a “cut-and-paste” mechanism (Matasci et al., 2011b) (Figure 2).

### 3.2 Type of Transposon Vector and PiggyBac Transposon Vector

A series of individual DNA transposons from different donor organisms have been identified in detail, including the *hAT* gene family element-Tol2 from the medaka fish (Kawakami et al., 2000), the engineered Tc1/mariner transposon, “*Sleep Beauty*” (SB) (Mikkelsen et al., 2003), and the insect-derived natural element *PiggyBac* (PB) (Tschorn et al., 2020; Rajendran et al., 2021). Although PB, Tol2, and SB do not show a preference against specific host cell chromosomes, they differ in their phylogenetic origin, biochemical properties, size of integrated target genes, and DNA sequence preference for translocation (Ding et al., 2005; Huang et al., 2010; Li et al., 2011; Meir et al., 2011; Wang et al., 2014). Tol2 and PB favor certain specific genomic regions and both insert mainly the upstream of the transcription start sites, CpG islands, and DNase I hypersensitive sites (Huang et al., 2010). In contrast, SB exhibits an almost stochastic integration. But it still shows a weak tendency for inserting in the transcribed regions and upstream regulatory sequences in mammalian cells (Gogol-Doring et al., 2016).

In terms of the efficiency of recombinant cell generation, the PB and SB systems were superior to the Tol2 system (Balasubramanian et al., 2016; Sato et al., 2020). Stable cell lines obtained by PB transposons are more efficient and have higher yields than those generated by common methods. Moreover, PB transposition seems to be largely independent from the host cell, as it can be performed *in vitro* with purified PB transposase and DNA elements (Burnight et al., 2012). Like retroviruses, SB and PB appear to utilize the barrier-to-autointegration factor to facilitate the insertion of transposon into the host genome by preventing transposon reintegration into the original vector (Wang et al., 2014). Thus, the PiggyBac transposon system has emerged as an attractive tool for target gene integration in mammalian cells (Balasubramanian et al., 2015). It is also a valuable and practical alternative to conventional target gene integration and can effectively generate clonal cell lines with stable or even enhanced expression (Matasci et al., 2011a). Aside from the high transposable activity in mammals, the PB system has been commercialized due to its inherent excellent properties, attracting researchers from different fields, and has been used as a genetic tool for genetic screening, likely to be the most promising genetic tool for clinical applications.

### 3.3 Optimization of Transposon Vector

Vector systems derived from SB and PB are most commonly used for biotechnology applications. In a dual-vector transposon system, transposase and ITR-containing transposons are constructed into two separate vectors, and the GOI are transferred into the host cells by co-transfection in different ratios. The transposase-SB100X in the optimized SB system showed a 100-fold increase in activity compared to the wild-type transposase (Mates et al., 2009; Voigt et al., 2016). The transposase of PB (mPB) showed a 20-fold increasing in transposition efficiency by codon optimization and was used in mammalian cells for the first time (Cadinanos and Bradley 2007; Liang et al., 2009). Cui et al. increased the transposition efficiency fourfold by optimizing the ITR sequence of the SB transposon vector in human HeLa cells (Cui et al., 2002). Later, an attempt was made to improve the transposition efficiency of the SB vector by introducing additional point mutations in ITR, but unfortunately no further improvement was achieved (Scheuermann et al., 2019). Transposition efficiency varies greatly in different type of cells (Izsvak et al., 2000; Troyanovsky et al., 2016). And the transposition efficiency and vector copy number (VCN) can be improved by optimizing the co-transposition ratio of transposase and transposon (Grabundzija et al., 2010). A phenomenon known as overexpression inhibition (OPI) would arise due to the excessive accumulation of transposase and eventually would prevent the efficient transposition. It has been proposed that high concentrations of transposase might saturate DRS in ITRs, slow down the synapse formation at transposon terminals (Liu and Chalmers 2014). For SB, the transposase-to-transposon co-transposition ratio ranges from 1:5 to 1:30, whereas PB transposase is not affected by OPI (Wilson et al., 2007).

In addition to the ratio of transposase and co-transfer that can affect transposition efficiency, direct optimization of the transposon donor can also improve transposition efficiency. In PB and SB, although PB could transpose linear DNA, its efficiency was much lower than circular DNA, while SB could not even transpose linear DNA. Therefore, linear donor DNA was not suitable for PB and SB. Several methods were also proposed to enhance the transposition efficiency, for example, linear DNA circularization in transfected cells using a recombinase (Yant et al., 2002; Nakanishi et al., 2011). It was found that two monocistrons which were applied to express the heavy and light chains separately were more advantageous than polycistron containing IRES or Furin-2A elements during the expression process of monoclonal antibodies with PB. The possible reason was that 2A elements affected the quality of expressed monoclonal antibodies through inappropriate cleavage, whereas conventional IRES lead to insufficient antibody expression level (Ahmadi et al., 2017). Insertion of MAR 1-68 into the center of the PB transposon does not affect transposition efficiency, whereas insertion into the edge of the transposon near the ITR side might interfere with transposase function and affect transposition efficiency. This is possibly due to the function alteration of transposase in targeting genomic locus and integrating the transposon sequence caused by MAR 1-68 (Ley et al., 2013). The addition of the 59-HS4 chicken b-globin (cHS4) insulator sequence in retina pigment epithelium cells leads to increased transgene expression levels for SB and PB vectors, respectively, but the cHS4 insulator did not show a long-term protective effect against the transgene silencing in retinal pigment epithelium cells (Sharma et al., 2012).

Different transposon systems show a weak bias towards certain specific genomic regions through screening transposon insertion sites, but cannot realize targeted integration. Kettlun et al. constructed a chimeric ZFP-piggyBac transposase displaying strong gene transfer activity by fusing a synthetic site-specific zinc finger protein (ZFP) to the N-terminal end of PB transposase. This manipulation capacitated the transposon system in targeted integration by combining the “cut-and-paste” mechanism of the transposon system with the target recognition property of ZFP, and successfully realized the targeted integration of ZFP-PiggyBac compared with the natural PiggyBac (Kettlun et al., 2011). After ZFP, the next generation of CRISPR gene targeting technology has become a hot research topic. In 2021, Bire et al. co-location the transposase and transposon near transcriptionally active rDNA copies using a nucleolar localization signal (NoLS). They found that nucleolus targeting increased transposition efficiency and mean transgene copy number, while targeting the 18S-coding region in the rDNA loci using a NoLS-FokI-dCas9 endonuclease, also can produce the targeted integrations, but this transgene expression level was lower compared to the PB transposase, indicated that the NoLS-coupled PB transposase and transposon can achieve more efficient integration (Bire et al., 2021).

More attentions have been paid to how to further optimize the transposable subsystem, not the problem of the transposon system itself. Given the endogenous transposases are also present in the target cells and there is no lack of transposase recognition sequences in the genome, we believe that either endogenous or exogenous transposases might have potential adverse influence on the cellular genome and GOI. However, it has been demonstrated that the introduction of exogenous transposase did not result in genome breakage and reorganization in the host cell and that the cell’s own transposase also did not destroy the successfully integrated GOI (Saha et al., 2015). However, integration of the backbone DNA of transposon and transposase vectors with the cellular genome still exists, and affect the normal expression of endogenous genes in the host cell. To solve this problem, weak promoters could be applied to reduce the expression level of transposase and shorten the plasmid backbone DNA size or integrating a suicide gene within the plasmid backbone to eliminate cells with backbone DNA integration.

Cell type, co-transfection ratio of transposon and transposase, transfection efficiency, and expression regulatory elements in the vector are all factors influencing the transposition efficiency of different transposon systems (Table 1). Only when all the aspects are fully considered can the transposition efficiency be maximized.

**TABLE 1:** Optimization of transposon vectors.

Classification	Method	Results	References
Optimization of transposase	Optimization of SB transposase	SB100X was produced, and the enzyme activity was increased 100-fold	Mates et al. (2009), Voigt et al. (2016)
	Codon optimization for PB transposase	Transposable efficiency increased by 20 times	Cadinanos and Bradley (2007), Liang et al. (2009)
Optimize the co-transfer ratio of transposase to transposon	-	Excess transposase prevents effective transposition. For SB, the co-transfer ratio is 1:5 to 1:30, while PB transposase is not affected by OPI.	Wilson et al. (2007)
Optimization of transposon vector	Comparing the transposition efficiency of linear and circular DNA	The transposition efficiency of linear DNA is much lower than that of circular DNA.	Yant et al. (2002), Nakanishi et al. (2011)
	Monoclonal antibodies were expressed using two monocistrons and polycistron mRNAs, respectively	Using two monocistrons to express the heavy and light chains separately is more advantageous than using polycistron containing IRES or 2A elements for simultaneous expression	Ahmadi et al. (2017)
	Combining MAR elements with transposon vectors	Insertion of MAR 1-68 into the transposon edge near the ITR may alter the mechanism of transposase targeting to genomic loci to integrate transposon sequences and affect transposition efficiency	Ley et al. (2013)
	Incorporation of cHS4 insulator sequence on transposon vector	Increased expression levels of target genes in SB and PB vectors	Sharma et al. (2012)
	ZFP fused with PB transposase	Formation of ZFP-PiggyBac fusion transposase with gene transfer activity and successfully achieved targeted integration of ZFP-PiggyBac compared with natural PiggyBac	Kettlun et al. (2011)
	Targeting transposase and transposon to ribosomal DNA (rDNA)	Increased transposition efficiency and increased the mean transgene copy number	Bire et al. (2021)
	Shortening the plasmid backbone or adding suicide genes to the plasmid backbone	Prevents integration of transposon plasmid backbone and host cells, affecting endogenous gene expression in host cells	Saha et al. (2015)

## 4 Transposon Vector Systems Used in Mammalian Cells

### 4.1 Improve Establishment Rate

Typically, GOI can only be inserted into the genome of mammalian cells by classical random integration manner, requiring a complex antibiotic-resistance screening step to obtain monoclonal cell lines. Moreover, the expression of GOI is often also influenced by the genomic DNA elements near the random insertion site, leading to significant variation in transcription level (Henikoff 1992; Martin and Whitelaw 1996; Narlikar et al., 2002). In addition, randomly integrated gene fragments are often inserted as multiple fragment tandems which results in the fragment duplication causing gene silencing (Garrick et al., 1998; McBurney et al., 2002). It follows that obtaining a cell line with a high level of protein expression usually requires time-consuming and laborious construction steps.

To address the drawbacks of classical integration methods, researchers have developed dihydrofolate reductase/methotrexate based gene amplification method (Kaufman 1990), as well as new manner to integrating GOI to the transcription active sites with the help of site-specific recombinases Flp, Cre and PhiC31 (Birling et al., 2009). However, these methods do not eliminate differences in cell-specific productivity, simplify the cell cloning step, and remove the limitations on integration efficiency and recombination sequence.

Transposon vector systems usually require only the successful screening of a pool of stably expressing cell lines (Balasubramanian et al., 2016) which are able to reduce the time required for the protein of interest production from 3–9 months to approximate 6–8 weeks (Rajendra et al., 2017). Among them, PB transposase has a lower requirement against recombination sequence, can efficiently insert fragments of up to 14 kb (Ding et al., 2005). Besides, it has the ability to insert up to 15 DNA fragments per cell with each fragment equally distributed across the genome (Wang et al., 2008; Rad et al., 2010) which greatly reduces the positional effects. In terms of gene manipulation, highly expressed cell lines maintaining a stable high expression level for at least 2 months could be constructed using this PB transposon system through a single transfection/screening step, greatly simplify the time-consuming cloning step. The 14 tested proteins were stably expressed in CHO-K1, HEK-293T, HEK-293F mammalian cell lines and the HEK-293S GnTI- cell line that is more difficult to stably transfect which were rapidly constructed with PB transposase (Li et al., 2013). And the feasibility for the production and purification of an endoplasmic reticulum-resident fucosyltransferase, a vascular endothelial growth factor Trap, and an anti-human epidermal growth factor receptor two single-chain variable-domain antibody using this system was also validated. In 2021, in the context of the COVID-19 pandemic, Agostinetto et al. rapidly constructed the high-titer stable pools in CHO cells using the Leap-in transposase® system to manufacture an anti-SARS-CoV2 monoclonal antibody in large scale. This greatly accelerated early clinical drug development and reduced the production period from 12–14 months using traditional method to 4–5 months (Agostinetto et al., 2021).

In addition, cell sorting is also a way to quickly obtain high expression cells. Matabaro et al. expressed glycosylphosphatidylinositol (GPI) anchoring protein in HEK293 cell line, while PGAP2 gene determines whether GPI can be secreted or not. First, endogenous PGAP2 was knocked down in HEK293 using CRISPR/Cas9 technology to construct the PGAP2-KO cell line, and then the exogenous PGAP2 gene was integrated into PGAP2-KO by establishing the PB transposon system. So, then the target protein could be expressed in the cell membrane to sort the highly expressed cells. Finally, the target protein can be secreted into the culture for subsequent isolation and purification after removing the PGAP2 gene by adding the transposase again (Matabaro et al., 2017). The flexible use of the transposon system’s ability to both integrate and shear genes allows researchers to artificially regulate whether target proteins are secreted or not. Therefore, the application of transposons is not only in gene integration, but in a larger scope of applications.

### 4.2 Enhance Recombinant Protein Expression

Matasci et al. first used PB vector to express a tumor necrosis factor receptor: Fc fusion protein (TNFR-Fc). In CHO cells, transposase and transposon were co-transformed at a ratio of 1:9, and then a pool of stably expressed cells were obtained through screening with different concentrations of puromycin. The results showed that the protein yield of the cell pool obtained by screening with high concentration of puromycin was higher. The increased productivity correlated with the GOI copy number per cell by RT-qPCR analysis, suggesting that cell lines showing high efficiency in protein production can be established using PB vectors and stringent screening process (Matasci et al., 2011b).

Balasubramanian et al. expressed TNFR-Fc in PB using CHO DG44 cells as hosts. The yield of TNFR-Fc reached 600 mg/L after 14 days of batch culture in a 1 L culture system. And the volumetric productivity of the cell pool can be maintained for up to 3 months in the absence of selecting pressure. This indicates that the production of recombinant proteins could be successfully improved using PB transposons (Balasubramanian et al., 2015). To further explore the role of transposon vector systems in recombinant proteins expression (Balasubramanian et al., 2016), Balasubramanian et al. performed a comprehensive comparison of three transposon systems (PB, Tol2 and SB) expressing TNFR-Fc. First, the effect of the number of helper vectors (transposase) on the productivity of the cell pool volume was investigated. For all three transposon systems, the productivity of TNFR-Fc increased with the number of helper vectors until reaching a plateau. Considering the efficiency of transgene integration, the optimal donor/helper vector ratio for the three transposon systems was determined to be 9:1 (w/w). In addition, in terms of stability of the volumetric yield of the cell pool, although it gradually decreases to about 50% of the initial level within 60 days, it is still 3-4 times of that obtained using the transposon-free cell pool. Finally, the volumetric yields of TNFR: Fc reached up to 1.3–1.5 g/L after 14 days of fed-batch culture with monoclonal cell lines constructed using the three transposon systems, indicating the positive effect of antibiotic -resistance screening in sustaining protein expression at high level.

### 4.3 Enhance Recombinant Antibody Expression

Barnard et al. used the PiggyBac transposon vector to express four antibodies in a CHO (CHO K1SV GS Knockout) cell line (Rajendra et al., 2016). The titers of four antibodies in the cell pools ranged from 2.3 to 7.6 g/L after 16 days of fed-batch cultures, increasing 4 to 12-fold compared with the control. To further investigate the reasons for the increased antibody expression levels of the PB transposon system, the mean GOI copy number of PB cell pools expressing two differentially expressed antibodies and the mRNA transcript levels of antibody heavy chain (HC) and light chain (LC) wer examined by RT-qPCR (Rajendra et al., 2017). The results demonstrated that the increased yield of antibodies in PB cell pools resulted from a combination of increased mean GOI copy number and transcription level. Subsequently, they assessed the quality of the antibodies obtained using the PB transposon system. The analyses result of analytical size exclusion chromatograph (aSEC), capillary electrophoresis—sodium dodecyl sulfate (CE-SDS) and capillary isoelectric focusing (CE-iCE) showed that no significant differences were observed compared with the control and the glycosylation patterns were very similar. And there was no difference in the density and viability of PB cell pool cells compared with the control. The titer of monoclonal antibody after 14 days cultivation was 4.8 g/L, which was about 2.5 times higher than that of the control. This indicates that the transposon system can improve the yield without affecting the quality of the antibody, providing a new way for clinical drug development.

## 5 Summary and Outlook

In recent years, mammalian cells have played an irreplaceable role in RTPs expression, and transposon vector systems which could improve yields without affecting the quality of RTPs deserve to be studied in greater depth. Compared with conventional expression vector systems, transposon system enables rapid construction of protein-expressing cell lines and can increase antibody yield without compromising quality. It also can shorten the production period for monoclonal antibodies against the SARS-CoV2 and accelerate the development of clinical drugs.

Besides, insertion of transposon vectors into matrix attachment regions could enhance the gene expression and insertion of cHS4 DNA could prevent the gene silencing phenomenon from affecting the target gene. To provide the transposon system with the ability to target integration, coupling the specific nucleolar localization signal NoLS with the transposon and transposase allows GOI to be localized to the rDNA-containing nucleoli, or fusing of the ZFP with the transposase enables the transposon system to have the ability to integrate in specific genomic regions. Other than that, DNA binding domains such as Gal4 DBD, Rep protein of adeno-associated virus, and TALE have been associated with the piggyBac transposase with the goal of targeting various chromosomal loci, with varying success. Unlike ZFNs and TALENs, CRISPR/Cas9 system does not rely on the recognition between the protein and the target DNA. The ribonucleotide complex is formed between the guide RNA and the target DNA. The GOI is recognized by ribonucleotide, then cleaved by Cas enzyme, and the DNA chain is repaired by homologous repair, combining transposon system with CRISPR/Cas9 technology, can establish a cell sorting method that allows proteins to be localized in the membrane to select highly expressed cells, also can target integrations based on Nols-FokI-dCas9 endonuclease coupled to NolS, although has lower integration efficiency compared to PB system coupled to NolS, CRISPR/Cas9 technology is still worth being investigated for transferring some specific GOI or specific loci. The combination of CRISPR/Cas9 and transposon systems can overcome their respective limitations, which will have high specificity and efficient integrative capacity double effects. It remains to be explored whether combining transposon vectors with CRISPR/Cas9 technology will bring more significant advantages in protein expression using mammalian cells.

The advantages of using transposon systems in RTPs production are obvious. It overcomes the limitations of conventional stable transfection and has practical value for the development of novel biological drugs and their industrial production in the future. In addition, it has been shown that transposon system can be used for gene therapy and cancer gene screening. Nevertheless, the understanding of transposon vectors is still at the research stage, there are still some problems to be solved. Although neither exogenous nor endogenous transposases affect the host cell genome and the successfully integrated GOI, the transposon vector backbone might still integrate with the genome of host cell and affect the normal expression of endogenous genes. The disadvantage using transposon vector system for recombinant protein production is the random integration of transgene, if inserted into the structural gene, it may lead to transcriptional read-through, insertional mutagenesis, structural changes and other negative effects. To further enhance RTPs expression and meet industrial production requirements, even to expand the application of transposon in gene therapy, transposon vectors are required to be optimized by investigating their interaction with multiple genetic elements and other technologies.
